# Anticoagulant treatment in German family practices – screening results from a cluster randomized controlled trial

**DOI:** 10.1186/s12875-014-0170-0

**Published:** 2014-10-25

**Authors:** Lisa-R Ulrich, Karola Mergenthal, Juliana J Petersen, Ina Roehl, Sandra Rauck, Birgit Kemperdick, Sylvia Schulz-Rothe, Andrea Berghold, Andrea Siebenhofer

**Affiliations:** Institute of General Practice, Goethe-University Frankfurt, Frankfurt, Germany; Institute for Medical Informatics, Statistics and Documentation, Medical University of Graz, Graz, Austria

**Keywords:** Oral anticoagulation, Family practice, Cluster randomization, Health services research

## Abstract

**Background:**

Oral anticoagulation (OAC) with coumarins and new anticoagulants are highly effective in preventing thromboembolic complications. However, some studies indicate that over- and under-treatment with anticoagulants are fairly common. The aim of this paper is to assess the appropriateness of treatment in patients with a long-term indication for OAC, and to describe the corresponding characteristics of such patients on the basis of screening results from the cluster randomized PICANT trial.

**Methods:**

Randomly selected family practices in the federal state of Hesse, Germany, were visited by study team members. Eligible patients were screened using an anonymous patient list that was generated by the general practitioners’ software according to predefined instructions. A documentation sheet was filled in for all screened patients. Eligible patients were classified into 3 categories (1: patients with a long-term indication for OAC and taking anticoagulants, 2: patients with a long-term indication for OAC but not taking anticoagulants, 3: patients without a long-term indication for OAC but taking an anticoagulant on a permanent basis). IBM SPSS Statistics 20 was used for descriptive statistical analysis.

**Results:**

We screened 2,036 randomly selected, potentially eligible patients from 52 family practices. 275 patients could not be assigned to one of the 3 categories and were therefore not considered for analysis. The final study sample comprised 1,761 screened patients, 1,641 of whom belonged to category 1, 78 to category 2, and 42 to category 3. INR values were available for 1,504 patients of whom 1,013 presented INR values within their therapeutic ranges. The majority of screened patients had very good compliance, as assessed by the general practitioner. New antithrombotic drugs were prescribed in 6.1% of cases.

**Conclusions:**

The screening results showed that a high proportion of patients were receiving appropriate anticoagulation therapy. The numbers of patients with a long-term indication for OAC therapy that were not receiving oral anticoagulants, and without a long-term indication that were receiving OAC, were considerably lower than expected. Most patients take coumarins, and the quality of OAC control is reasonably high.

**Trial registration:**

Current Controlled Trials ISRCTN41847489.

## Background

Oral anticoagulation (OAC) with coumarins or the new oral anticoagulants (NOACs) dabigatran, rivaroxaban and apixaban, has been shown to be highly effective in preventing thromboembolic complications [1]. In German family practices, the most common indications for long-term OAC therapy are atrial fibrillation/flutter, recurrent venous and/or pulmonary thromboembolisms, and mechanical heart prostheses [2]. However, some studies indicate that over- and under-treatment with anticoagulants are fairly common. According to the Registry of the German Competence NETwork on Atrial Fibrillation (AFNET), which includes over 9,000 patients, 72% of eligible patients received anticoagulation treatment, while 17% were only given antiplatelet drugs, and as many as 11% of patients indicated for anticoagulation received no therapy whatsoever [3]. A survey by the West Birmingham Atrial Fibrillation Project indicated that of 71 patients who were not treated with warfarin, only 17% had any recorded contraindications [4]. A study in 16 German family practices showed that there is room for improvement in monitoring, documentation quality and patient participation in anticoagulant treatment [5]. OAC self-management, in which patients self-monitor their INR (International Normalized Ratio) values and self-adjust their medication dosages, is a possible option to increase the involvement of patients in their antithrombotic treatment. Studies confirm that thromboembolic events occur less frequently in patients performing self-management [6,7], and this is also true of elderly patients [8].

However, little is known about the quality of routine anticoagulation therapy in German family practices. Since March 2012, the Institute of General Practice, Goethe-University Frankfurt, Germany, has therefore been conducting the cluster randomized controlled PICANT trial (Primary Care Management for Optimized Antithrombotic Treatment), primarily in family practices in the state of Hesse, Germany, on patients with a long-term indication for OAC [9]. The aim of the PICANT study is to find out whether a best practice model that includes major elements of case management and patient education, such as self-management, can improve antithrombotic management in primary health care in terms of reducing major thromboembolic and bleeding events over a follow up period of 2 years.

The aim of this paper is to assess treatment with anticoagulants in German family practices on the basis of the screening results of the PICANT trial by determining to what extent over- and under-treatment exist, finding out how many screened patients have INR values within their therapeutic target ranges, and by describing the distinguishing characteristics of screened patients with a long-term indication for OAC. The manuscript adheres to the STROBE Statement/Checklist [10].

## Methods

Practices and patients were recruited between June 2012 and December 2012. The study was approved in June 2012 by the ethical review committee of the University Hospital, Goethe-University Frankfurt, Germany.

### Practice recruitment

The recruitment process has been described in detail elsewhere [9]. In brief, we identified potentially eligible practices from a list provided by the Association of Statutory Health Insurance Physicians (mandatory registration). Practices were stratified according to the number of inhabitants in the postal area in which the practice was located. The address database was broken down into groups according to the physician’s specialization (general practitioner (GP), specialist in internal medicine, or medical practitioner). As the registration list only contains the names and addresses of family doctors, we mailed information on the trial to 568 randomly selected practices (6% of all registered practices in 2012) and invited them to participate. Inclusion criteria were only checked for those who were interested in participation. The appropriate sample size was calculated and practice recruitment stopped when 52 practices had enrolled, even though further practices were interested in participating.

### Patient recruitment

Each participating practice was visited after practice recruitment, but before cluster randomization, and asked to use the practice software to generate a screening list of potentially eligible patients on the basis of predefined instructions and search terms provided by the study team members [9]. The GPs then checked the lists and deleted eventual cases of patients that had only been seen occasionally or had died in the meantime. Inclusion criteria were then assessed by the GP and study team for 30 randomly selected patients from the list. A documentation sheet was filled in for each screened patient. To avoid a selection bias, the order of the patients assessed for eligibility was chosen by means of the random number generator function in Microsoft Excel®.

### Data collection

The GP provided the following data for the screened patients: age, sex, long-term indication for OAC, antithrombotic medication, reasons for exclusion, most recent INR value and date of assessment in the family practice, and migration background. The GP was also asked to allocate each patient’s level of compliance to one of three categories (very good compliance, good compliance, non-compliant). The study team determined the INR target range for each individual patient according to current guideline recommendations [11,12]. On the basis of the data provided by the GP, the study team classified each screened patient in one of three categories (1: patients with a long-term indication for OAC and taking anticoagulants, 2: patients with a long-term indication for OAC but not taking anticoagulants, 3: patients without a long-term indication for OAC but taking an anticoagulant on a permanent basis). Category 1 represents patients receiving appropriate OAC therapy, category 2 patients that are under-treated, and category 3 patients that are over-treated.

In agreement with study protocol, only patients from categories 1 and 2 were eligible for study participation [9]. The 30 identified patients from each practice were then sent a letter of invitation to participate in the trial, and patient recruitment discontinued when 15 patients per practice had agreed in writing to participate. After successful patient recruitment, the family practices were randomly allocated to the best practice model (intervention group) or routine care (control group) in a ratio of 1:1, using the web-based randomization service Randomizer for Clinical Trials (www.randomizer.at). IBM SPSS Statistics 20 was used for descriptive statistical analysis.

## Results

**Figure 1:**
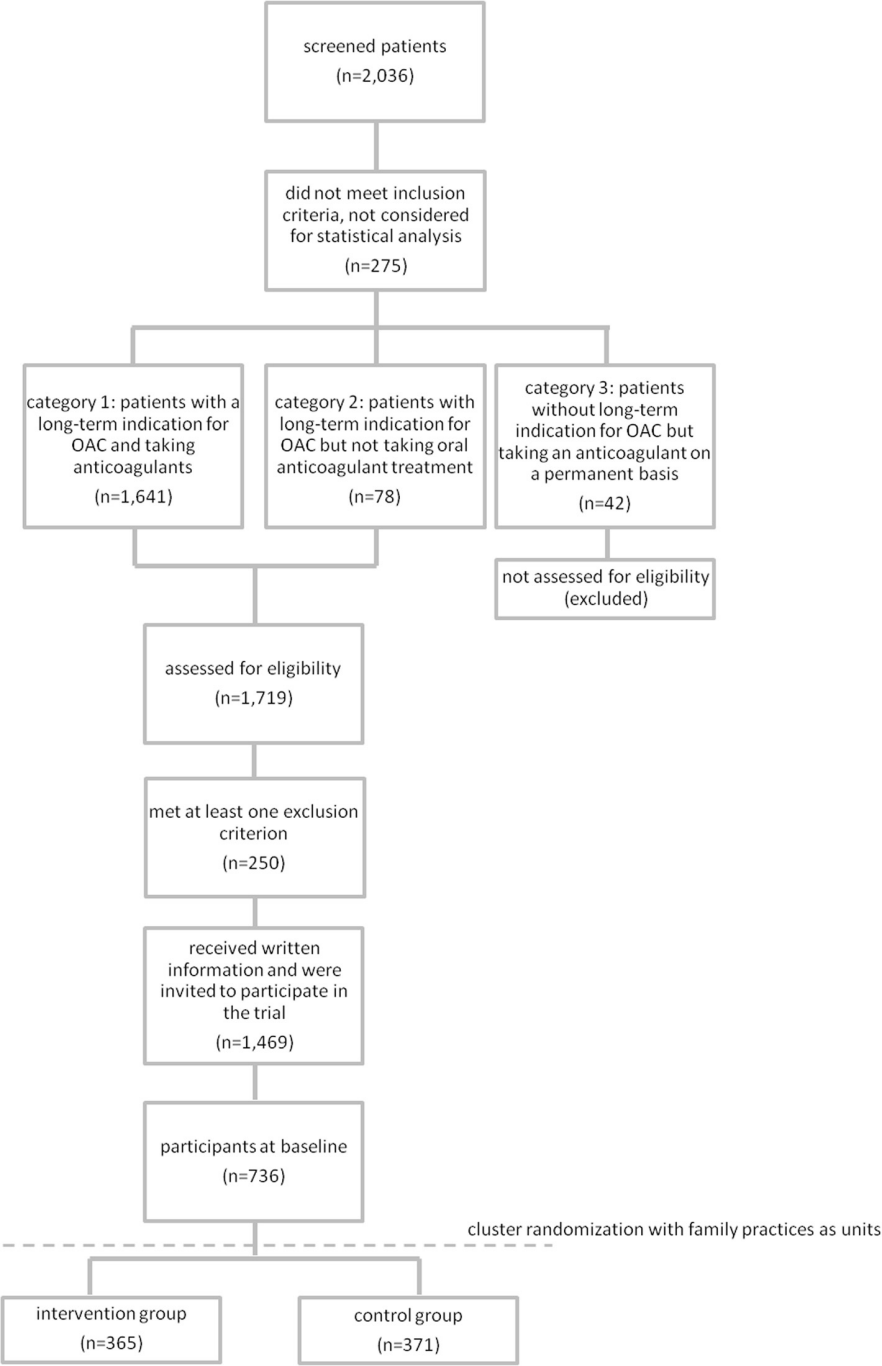
**The screening process in detail and the results of the screening.**

We screened 2,036 potentially eligible patients from 52 family practices. Of these, 275 did not meet the inclusion criteria (e.g. patients with a single thromboembolic event with low risk of recurrence and no long-term indication for OAC, and patients with arterial disease (e.g. peripheral artery disease) who were not eligible for study participation). The final study sample comprised 1,761 patients. The mean age of the patients was 74.6 years (SD 10.4) and 52.7% of them were male. Figure 1 shows the screening process in detail.

1,641 (93.2%) of the screened patients were receiving appropriate therapy, i.e. they had a long-term indication for OAC and received it (category 1). A further 78 (4.4%) were under-treated (category 2), i.e. they had a long-term indication for OAC but received no corresponding therapy. The majority (94.9%) of patients in category 2 had atrial fibrillation / flutter with a CHA_2_DS_2_VASc-Score of >1 for which therapy with OAC is recommended [13]. Three patients had recurrent deep venous thromboembolism and one a pulmonary embolism with a previous episode of thromboembolism.

The remaining 42 (2.4%) screened patients were over-treated, i.e. they took an anticoagulant on a permanent basis despite having no long-term indication for it (category 3). Examples of ‘over-treated’ patients were those that previously had deep venous thromboembolism with a low risk of recurrence and for whom treatment could have been discontinued. Most category 3 patients were taking coumarins (95.2%), with only two on NOACs (1 dabigatran, 1 rivaroxaban). While assessing patients for their eligibility, we recommended to GPs that he or she perform a reappraisal to check whether the patient’s indication had expired.

The main indication for OAC was atrial fibrillation/flutter (1,415 patients, 80.4%). Most patients (1,554, 88.2%) were receiving coumarins, while 107 (6.1%) were taking NOACs (46 dabigatran, 61 rivaroxaban). 149 patients (8.5%) performed OAC self-management (measured INRs and adjusted doses themselves) and 1 patient tested himself but received help in adjusting the dose. According to the GPs, 160 (9.1%) of the screened patients had a migration background. According to their GPs, the majority (65.7%) of screened patients demonstrated very good compliance, and only 7.3% were regarded as non-compliant. INR values were available for 1,504 patients, of whom 1,013 (67.3%) patients presented values within the therapeutic target ranges recommended in guidelines. Table 1 shows patient characteristics in detail.

**Table 1:** **Characteristics of screened patients**

**Patient characteristics**	**n = 1,761 (%)**
Mean age years (SD)	74.6 (10.4)
Sex	
*Male*	928 (52.7%)
*Female*	833 (47.3%)
Long-term indication for oral anticoagulation therapy	
*Atrial fibrillation/flutter*	1,415 (80.4%)
*Recurrent venous thromboembolism*	142 (8.1%)
*Recurrent pulmonary embolism*	37 (2.1%)
*Mechanical heart prosthesis*	93 (5.3%)
*Intracardiac thrombus*	10 (0.6%)
*Other indication*	22 (1.2%)
*No long-term indication*	42 (2.4)
Antithrombotic medication*	
*Coumarin*	1,554 (88.2%)
*Dabigatran*	46 (2.6%)
*Rivaroxaban*	61 (3.5%)
*Antiplatelet therapy*	55 (3.1%)
*Other antithrombotic medication*	11 (0.6%)
*No antithrombotic medication*	34 (2%)
Within therapeutic INR target range**	1,013 (67.3%)
Patient self-management	
*INR self-measuring and dose adjustment*	149 (8.5%)
*INR self-testing and dose adjustment by GP*	1 (0.1%)
Patients with migration background	160 (9.1%)
Patients compliance assessed by GP	
*Very good compliance*	1,157 (65.7%)
*Good compliance*	450 (25.6%)
*Non-compliant*	129 (7.3%)
*No assessment available*	25 (1.4%)

*Apixaban had not been approved at the time of screening.

**INR measurements available for 1,504 patients (89% of patients from category 1 (appropriate OAC treatment) and 3 (over-treatment with oral anticoagulants)).

All patients in categories 1 and 2, i.e. a total of 1,719 (97.6%), were initially considered eligible for study participation, but 250 (14.5%) of them were later recognized as meeting at least one exclusion criterion and did not participate in the trial (as shown in Table 2). The main reason for exclusion was dementia (44.4%). In the end, 1,469 (83.4%) of the original group of patients received written information and were invited to participate in the trial. 52 practices and 736 patients were randomized into the study.

**Table 2:** **Number of patients excluded**

**Reason for exclusion**	**n (%)**
Dementia	111 (44.4%)
Life expectancy <6 months	21 (8.4%)
Lack of German language skills	20 (8.0%)
Residence in nursing home or residential care home	14 (5.6%)
Psychosis	5 (2.0%)
Severe sight disorder or auditory defect	9 (3.6%)
Alcohol or drug abuse	5 (2.0%)
Other reasons for exclusion	65 (26.0%)
**Total**	**250 (100%)**

## Discussion

The screening results of the PICANT trial revealed that over 90% of the mainly elderly patients received appropriate long-term oral anticoagulation that was within their therapeutic INR target ranges over 65% of the time. Patients on new antithrombotic medications were rare when the study began, and self-management of OAC was performed by fewer than 10% of patients.

Our findings are consistent with those of a study by Nilsson et al. that aimed to assess warfarin treatment in Swedish primary health care centres [14]. Patient characteristics such as age, sex, and the primary indications for treatment (firstly atrial fibrillation and secondly deep venous thrombosis) were comparable. However, in contrast to Nilsson et al. we also included patients taking antiplatelet drugs and NOACs which had not been approved in early-2000 when Nilsson’s study was conducted. As part of PICANT, we will follow up on our patients over a period of 2 years and expect to see a shift from the old to the new drugs. In Germany, the numbers of prescriptions for the NOACs dabigatran and rivaroxaban increased sharply in 2012, rising almost 400% in the case of dabigatran and more than 1,000% for rivaroxaban [15]. However, further research into the use of NOACs under practice conditions is needed [16]. The reasons why GPs are encouraging patients to switch to the new antithrombotic drugs (dabigatran, rivaroxaban, apixaban) will be examined at the end of the trial [9].

In contrast to several other studies, the number of patients receiving appropriate OAC therapy was high. As mentioned in the introduction, under- and over-treatment were found to be extremely common in the AFNET trial [3] and the West Birmingham Atrial Fibrillation Project [4]. In addition, a systematic review by Ogilvie et al. discovered that patients with atrial fibrillation and a high risk of stroke were under-treated with oral anticoagulants in the majority of identified studies [17]. In our study only about 4.4% of patients were under-treated, and most of those had atrial fibrillation with a CHA_2_DS_2_VASc-Score >1. The percentage who were over-treated and had no further indication for OACs was also low (2.4%). We suspect that one of the main reasons for over- and under-treatment among patients is that some patients do not regularly attend the practice and that necessary changes to their treatment are simply overseen, either due to higher risk (possibly as a result of increasing age, or new co-morbidities), or because the initial indication has expired. A current UK study on the use of anticoagulants in family practices found that one third of patients with atrial fibrillation who were indicated for oral anticoagulation do not receive any such therapy [18]. However, it should be borne in mind that good reasons sometimes exist not to treat patients with oral anticoagulants (e.g. contraindications, bleeding complications, or a high risk of falls, especially in elderly patients). Over 67% of the patients we screened had INR values (based on only one measurement) within their therapeutic INR target ranges, which is consistent with a systematic review written by Wan et al. that presents results from 6 randomized controlled trials [19]. Our INR screening results compare favourably with a German study conducted in 2007, which reported that only about 55% of patients with atrial fibrillation had INR values within their recommended target ranges [20].

Only about 8% of all screened patients with a long-term indication for OAC actually performed OAC self-management. As early as 2009, about 150,000 patients in Germany were self-monitoring their INR-values and self-adjusting their dosages accordingly [21]. On the assumption that about 1 million patients in Germany take coumarins [15], the proportion of patients performing self-management in our study is lower than in the German population as a whole. Self-management helps prevent thromboembolic complications [6,7], indicating that strategies aimed at increasing patient involvement may enhance chronic illness care [22]. Self-monitoring is also one important attribute of the intervention group in the PICANT trial [9], for which final results will be presented after study completion in March 2015.

This paper only presents one cross-sectional investigation in German family practices without any stratification of the German population as a whole. Nevertheless, we believe our sample is representative, as we randomly selected GPs and their patients in accordance with good clinical practice guidelines [9]. Dementia was the main reason for exclusion from our study, as expressed in a patient’s inability to provide informed consent. That may have led to some bias, but dementia alone is no reason to deny a patient anticoagulation therapy and these patients were still included in the screening results presented in this manuscript.

## Conclusions

The screening results show that most participants in the PICANT trial are receiving appropriate OAC therapy, and only a minority of patients taking oral anticoagulants are over- or under-treated. The proportion of inappropriately treated patients in our study is also much lower than reported in comparable studies, despite similar patient characteristics. From the onset of the study, the quality of anticoagulation therapies was high, and since the new oral anticoagulants have only recently been approved, most patients were still on coumarins.

## Abbreviations

AFNET: The central registry of the German competence NETwork on atrial fibrillation
GP: General practitioner
INR: International normalized ratio
NOACs: New oral anticoagulants
OAC: Oral anticoagulation
PICANT: Primary care management for optimized antithrombotic treatment
SD: Standard deviation

## References

[1] Guyatt GH: **Methodology for the development of antithrombotic therapy and prevention of thrombosis guidelines. Antithrombotic therapy and prevention of thrombosis, 9th ed: American college of chest physicians evidence-based clinical practice guidelines.** *Chest* 2012, **141:**53S. 10.1378/chest.11-2288 PMC3278053 22315256

[2] Hua TD, Vormfelde SV, Abed MA, Schneider-Rudt H, Sobotta P, Chenot JF (2010). Orale Antikoagulation in der Hausarztpraxis. [Oral Anticoagulation Therapy in Family Medicine]. Z Allg Med.

[3] Nabauer M, Gerth A, Limbourg T, Schneider S, Oeff M, Kirchhof P, Goette A, Lewalter T, Ravens U, Meinertz T, Breithardt G, Steinbeck G (2009). The registry of the German competence NETwork on atrial fibrillation: patient characteristics and initial management. Europace.

[4] Lip GY, Golding DJ, Nazir M, Beevers DG, Child DL, Fletcher RI (1997). A survey of atrial fibrillation in general practice: the West Birmingham atrial fibrillation project. Br J Gen Pract.

[5] Saal K, Hofmann B, Blauth E, Rohe J, Beyer M, Harder S, Gerlach FM (2009). Analyse des Behandlungsprozesses bei der oralen Antikoagulationstherapie zur Identifikation von Sicherheitsproblemen in der hausärztlichen Versorgung [Identifying safety problems in the General Practice - analysis of the treatment process of oral anticoagulation therapy]. Z Allg Med.

[6] Heneghan C, Ward A, Perera R, Bankhead C, Fuller A, Stevens R, Bradford K, Tyndel S, Alonso-Coello P, Ansell J, Beyth R, Bernardo A, Christensen TD, Cromheecke M, Edson RG, Fitzmaurice D, Gadisseur APA, Garcia-Alamino JM, Gardiner C, Hasenkam M, Jacobson A, Kaatz S, Kamali F, Khan TI, Knight E, Körtke H, Levi M, Matchar DB, Menéndez-Jándula B, Rakovac I (2012). Self-monitoring of oral anticoagulation: systematic review and meta-analysis of individual patient data. Lancet.

[7] Siebenhofer A, Jeitler K, Horvath K, Habacher W, Schmidt L, Semlitsch T (2014). Self-management of oral anticoagulation. Dtsch Arztebl Int.

[8] Siebenhofer A, Rakovac I, Kleespies C, Piso B, Didjurgeit U (2008). Self-management of oral anticoagulation reduces major outcomes in the elderly. A randomized controlled trial. Thromb Haemost.

[9] Siebenhofer A, Ulrich LR, Mergenthal K, Roehl I, Rauck S, Berghold A, Harder S, Gerlach FM, Petersen JJ: **Primary care management for optimized antithrombotic treatment [PICANT]: study protocol for a cluster-randomized controlled trial.** *Implementation Sci* 2012, **7:**79. 10.1186/1748-5908-7-79 PMC3499320 22929015

[10] von Elm E, Altman DG, Egger M, Pocock SJ, Gøtzsche PC, Vandenbroucke JP (2008). The strengthening the reporting of observational studies in epidemiology (STROBE) statement: guidelines for reporting observational studies. J Clin Epidemiol.

[11] Guyatt GH, Akl EA, Crowther M, Gutterman DD, Schünemann HJ (2012). Executive summary: antithrombotic therapy and prevention of thrombosis, 9th ed: American college of chest physicians evidence-based clinical practice guidelines. Chest.

[12] Hirsh J, Fuster V, Ansell J, Halperin JL (2003). American heart association/American college of cardiology foundation guide to warfarin therapy. Circulation.

[13] Camm AJ, Lip GYH, de Caterina R, Savelieva I, Atar D, Hohnloser SH, Hindricks G, Kirchhof P, Bax JJ, Baumgartner H, Ceconi C, Dean V, Deaton C, Fagard R, Funck-Brentano C, Hasdai D, Hoes A, Kirchhof P, Knuuti J, Kolh P, McDonagh T, Moulin C, Popescu BA, Reiner Z, Sechtem U, Sirnes PA, Tendera M, Torbicki A, Vahanian A, Windecker S (2012). 2012 focused update of the ESC guidelines for the management of atrial fibrillation: an update of the 2010 ESC guidelines for the management of atrial fibrillation* developed with the special contribution of the European heart rhythm association. Europace.

[14] Nilsson GH, Björholt I, Johnsson H: **Anticoagulant treatment in primary health care in Sweden - prevalence, incidence and treatment diagnosis: a retrospective study on electronic patient records in a registered population.** *BMC Fam Pract* 2003, **4:**3. 10.1186/1471-2296-4-3 PMC156632 12675952

[15] Schwabe U, Paffrath D (2013). Arzneiverordnungs-Report 2013: Aktuelle Daten, Kosten, Trends und Kommentare.

[16] Innasimuthu AL, Kumar S, Akter S, Borer JS (2013). New oral anticoagulants: great promise for therapeutic advance but great knowledge gaps remain to be filled. Cardiology.

[17] Ogilvie IM, Newton N, Welner SA, Cowell W, Lip GY (2010). Underuse of oral anticoagulants in atrial fibrillation: a systematic review. Am J Med.

[18] Cowan C, Healicon R, Robson I, Long WR, Barrett J, Fay M, Tyndall K, Gale CP (2013). The use of anticoagulants in the management of atrial fibrillation among general practices in England. Heart.

[19] Wan Y, Heneghan C, Perera R, Roberts N, Hollowell J, Glasziou P, Bankhead C, Xu Y (2008). Anticoagulation control and prediction of adverse events in patients with atrial fibrillation: a systematic review. Circ Cardiovasc Qual Outcomes.

[20] McBride D, Brüggenjürgen B, Roll S, Willich SN (2007). Anticoagulation treatment for the reduction of stroke in atrial fibrillation: a cohort study to examine the gap between guidelines and routine medical practice. J Thromb Thrombolysis.

[21] Braun S, Völler H, Soppa C, Taborski U (2009). Aktualisierte Leitlinie “Gerinnungsselbstmanagement”. Dtsch Med Wochenschr.

[22] Glasziou P, Irwig L, Mant D (2005). Monitoring in chronic disease: a rational approach. BMJ.

